# A Novel Pathogenic Variant of DICER1 Gene in a Young Greek Patient with 2 Different Sex-Cord Ovarian Tumors and Multinodular Goiter

**DOI:** 10.3390/ijms26051990

**Published:** 2025-02-25

**Authors:** Afroditi Roumpou, Argyro-Ioanna Ieronimaki, Aspasia Manta, Ioannis G. Panayiotides, Constantine A. Stratakis, Sophia Kalantaridou, Melpomeni Peppa

**Affiliations:** 1 Endocrine Unit, Second Propaedeutic Department of Internal Medicine, “Attikon” Hospital, School of Medicine, National and Kapodistrian University of Athens, 12462 Athens, Greece; 2 Second Department of Pathology, “Attikon” Hospital, School of Medicine, National and Kapodistrian University of Athens, 12462 Athens, Greece; anismed03@yahoo.gr (A.-I.I.); ioagpan@med.uoa.gr (I.G.P.); 3 Human Genetics & Precision Medicine, Institute for Molecular Biology & Biotechnology (IMBB), Foundation for Research & Technology Hellas (FORTH), 70013 Heraklion, Greece; 4 ASTREA Health: Precision Medicine and Longevity, 11528 Athens, Greece; 5 Medical Genetics, Henry Dunant Hospital, 11526 Athens, Greece; 6 Third Department of Obstetrics and Gynecology, “Attikon” Hospital, School of Medicine, National and Kapodistrian University of Athens, 12462 Athens, Greece; 7 Third Department of Internal Medicine, “Sotiria” General Hospital, School of Medicine, National and Kapodistrian University of Athens, 11527 Athens, Greece

**Keywords:** DICER1 syndrome, DICER1 gene, hyperandrogenemia, juvenile granulosa cell tumor, multinodular goiter, novel DICER1 variant, Sertoli–Leydig cell tumor

## Abstract

DICER1 syndrome (DICERs) represents a tumor predisposition genetic syndrome, inherited in an autosomal dominant manner. Germline loss-of-function variants of the *DICER1* gene lead to impaired processing of microRNA, gene expression, and increased risk of tumorigenesis. Although pleuropulmonary blastoma (PPB) is the hallmark of the syndrome, multiple extrapulmonary malignant and non-malignant conditions have also been described, including multinodular goiter (MNG) and sex-cord stromal tumors. MNG is one of the most common components and is associated with an increased risk of thyroid carcinoma. Sertoli–Leydig cell tumor (SLCT) represents the most prevalent type of sex-cord stromal tumor associated with the syndrome, whereas juvenile granulosa cell tumor (JGCT) is considered to be a very rare phenotype. They both may present with abdominal pain due to mass effect and menstrual irregularities in case of hormone production. Although they exhibit low rates of mortality, recurrence rates highly depend on the grade of malignancy. Herein, we report a novel pathogenic *DICER1* variant associated with MNG, bilateral ovarian SLCT, and JGCT in a young Greek patient. Clinicians should be aware of a potential germline *DICER1* variant when evaluating MNG in young patients, especially if it coexists with other neoplasms.

## 1. Introduction

**Figure 1:**
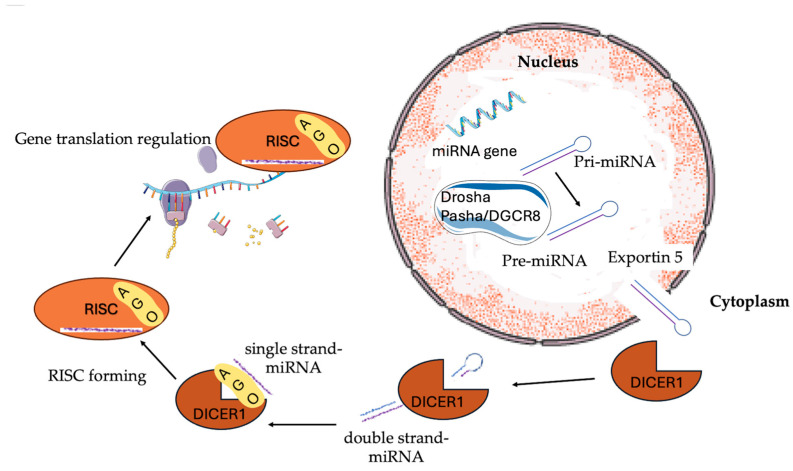
The role of DICER1 protein in the microRNA (miRNA) generation pathway. Initially, RNase II in the nucleus converts longer precursors from DNA into primary RNAs (pri-miRNAs). Pri-miRNA is broken down by Drosha and Pasha/DGCR8 (DiGeorge critical region 8) into pre-miRNA, which is a “hairpin structure”. After it is exported from the nucleus via exportin 5, 3p and 5p miRNAs are cleaved by the DICER1 protein, forming a short miRNA duplex molecule that is later degraded into a single-strand miRNA (mature miRNA). Then, one strand of the miRNA duplex is combined with other proteins, such as members of the Argonaute (AGO) family, to form an RNA-induced silencing complex (RISC) that targets and controls messenger RNA, regulating suppression of gene expression.

DICER1 syndrome (DICER1s) is a genetic syndrome characterized by the development of multiple benign and malignant neoplasms, as well as non-neoplastic conditions. A plethora of germline loss-of-function alterations in the homonymous gene results in impaired microRNA (miRNA) processing, altered gene expression, and tumorigenesis [1]. The *DICER1* gene is located at 14q32.13, and it encodes a multidomain endoribonuclease of 1922 amino acids that plays a pivotal role in the synthesis pathway of miRNA [2]. Specifically, RNA polymerase II transcribes microRNAs from pri-miRNAs, which are long RNA precursors [3]. Pri-miRNAs are processed in the nucleus by the RNA-binding protein Pasha/DGCR85 (DiGeorge critical region 8) and the RNase III enzyme Drosha to form pre-miRNAs that are folded into stem-loop structures [4,5]. After exporting the nucleus by exportin 5 [6], 3p and 5p miRNAs are cleaved from the miRNA precursor by the domains IIIa and IIIb of the ribonuclease DICER1 and, subsequently, produce the miRNA, a double-stranded RNA [7,8]. Additionally, DICER1 promotes the formation of the RNA-induced silencing complex (RISC), which includes other proteins, such as members of the Argonaute (AGO) protein family. One of the two complementary short RNAs gets incorporated into the RISC complex, which targets and regulates messenger RNA, by either promoting its destruction or disrupting its translation (Figure 1) [9,10,11,12].

DICER1s is caused by germline inactivating alterations in the *DICER1* gene, inherited with an autosomal dominant pattern, while a noteworthy percentage of all cases (10–20%) seem to arise de novo [13]. Deletions, duplications, insertions, transitions, and transversions are among the mutations found in *DICER1* [14]. The majority of patients developing DICER1-associated tumors, except for one hereditary germline *DICER1* variant, also have another acquired somatic missense *DICER1* alteration in one of the “hot-spot” codons included in the RNAse IIIb domain (E1813, D1705, D1709, D1713, and G1809) [2]. Moreover, approximately 10% of people with a predisposing *DICER1* variant are mosaic, meaning the somatic mutations are acquired during postzygotic development. Mosaic individuals are rather uncommon, but their detection poses a special challenge for germline testing, as only a portion of cells harbor the mutation [15,16]. It is considered that approximately 1:2500–1:10,600 individuals in the general population are heterozygotes for a pathogenic or likely pathogenic *DICER1* variant [17].

Since the first description of pleuropulmonary blastoma (PPB) in childhood by Manivel et al. [18], the term “pleuropulmonary blastoma familial tumor susceptibility syndrome” has been introduced until the identification of *DICER1* gene pathogenic variants as the underlying cause of the disorder [19]. DICERs may present with a vast variety of clinical phenotypes apart from PPB, including malignant tumors, such as blastomas (pituitary, pineal), ovarian Sertoli–Leydig cell tumor (SLCT), cervical embryonal rhabdomyosarcoma, Wilms tumor, renal sarcoma, thyroid carcinoma, mesenchymal hamartoma of the liver, and neuroblastoma [13,20,21,22]. The syndrome also involves some benign conditions, such as multinodular goiter (MNG), cystic nephroma, hamartomatous intestinal polyps, and nasal chondromesenchymal hamartoma [23,24]. Non-neoplastic disorders such as macrocephaly, kidney structural abnormalities, retinal abnormalities, dental perturbations, and the GLOW syndrome (global developmental delay, lung cysts, overgrowth, and Wilms tumor) have also been observed in people with germline *DICER1* variants [19]. Although they are at a higher risk of developing cancer, the majority of carriers with a germline *DICER1* variant may have healthy lives [10]. The conditions associated with DICERs are categorized regarding frequency and malignant potential in Table 1 and Table 2, respectively [25].

PPB is the most frequent manifestation and the main cause of mortality, while ovarian SLCT, MNG, and cystic nephroma are some of the most prevalent components [14,24,26]. Early-onset MNG has been strongly related to the syndrome. In fact, it has been reported that the incidence of MNG or thyroidectomy among carriers of *DICER1* germline pathogenic variants is 75% and 17% in women and men, respectively, before the age of 40 years [27]. In DICER1-related MNG, molecular analysis of the nodules has indicated that they are clonal, since they harbor a second somatic mutation, different for each nodule, in addition to the responsible germline alteration [2,28]. The risk for thyroid carcinoma is increased by over 16 times compared to healthy controls, with minimally invasive follicular thyroid carcinoma and the follicular variant of papillary thyroid carcinoma being the most frequently reported [27]. Generally, thyroid cancer is associated with a favorable prognosis, similar to that of sporadic differentiated thyroid carcinoma [13].

Neoplasms of the gynecologic tract, especially ovarian SLCTs, could be the first clinical manifestation of DICER1s running in a family and usually develop from childhood until adulthood [16]. SLCTs are an extremely rare type of ovarian sex-cord stromal tumors (about 1%) [29], accounting for less than 0.5% of all primary ovarian neoplasms [30,31]. However, SLCTs constitute the second most common tumors associated with DICER1s, after PPBs [32,33,34,35,36,37], with more than 50% of SLCT patients carrying a pathogenic variant [38]. They may be well, moderately, or poorly differentiated according to the World Health Organization (WHO) criteria [39]. Most SLCTs are well differentiated, have a favorable prognosis, and have rare recurrence, whereas less differentiated tumors may have a more aggressive disease course [29,40]. Although even advanced-stage SLCTs can have a positive prognosis due to their susceptibility to chemotherapy, 20% recur or develop potentially lethal metastases [41]. It has been supported that 97–100% of patients with intermediate or poorly differentiated SLCTs have a *DICER1* pathogenic variant, while this is noted in only 12% of those with well differentiated tumors [1,33,42]. Compared to sporadic SLCTs, patients with DICER1-associated SLCTs tend to have features associated with hyperandrogenemia due to hormone production, early presentation, and increased risk of recurrence [35,37]. The mortality rate with SLCTs in DICER1s is minimal, and fewer than 5% of recorded deaths are linked to SLCTs [10].

Granulosa cell tumors account for 1–5% of all ovarian tumors, arise from the granulosa cells of the ovarian follicle, and are divided into adult and juvenile types, with the latter being the minority of the cases, presenting in younger patients, usually <30 years old [43,44,45]. Clinical presentation usually includes abdominal pain and increasing abdominal girth, and in cases of hormonally active tumors (estradiol production), menstrual irregularities, or precocious puberty. Patients with juvenile granulosa cell tumors (JGCT) have a very stage-dependent prognosis, with a 97% survival rate in patients with stage 1 tumors that are limited to the ovary [16]. Herein, we present for the first time a case of a young Greek female patient with a history of non-toxic MNG and both a bilateral SLCT diagnosed at the age of 18 years and JGCT at the age of 21 years, due to a novel pathogenic *DICER1* gene variant.

## 2. Case Presentation

### 2.1. Multinodular Goiter (MNG)

In 2017, a 16-year-old Greek female patient was referred to our Endocrinology Unit for further investigation of an MNG found incidentally on thyroid ultrasonography. In particular, the imaging identified 7 nodules ranging from 5.5 to 25 mm in size. Fine needle aspiration (FNA) of the two larger nodules was reported as TBSII (The Bethesda system), according to the Bethesda classification system, being consistent with nodular thyroid hyperplasia. Thyroid function tests and calcitonin levels (3.3 ng/L, Ref: 1–10 ng/L) were unremarkable, and she was advised to regular follow-up.

### 2.2. Irregular Menstruation-Hyperandrogenemia

One year later (2018), at the age of 17 years, she was referred to a gynecologist due to irregular menstruation and acne. Menarche occurred at the age of 12 years, and until then, she did not mention any menstrual irregularities. Physical examination revealed normal breast and pubic hair development (Tanner stage V). The hormone profile revealed raised testosterone levels (3.1 ng/mL, Ref: 0.1–0.5 ng/mL), low follicle-stimulating hormone (FSH; 1.3 mIU/mL, Ref: 3–8.1 mIU/mL), and low normal luteinizing hormone (LH; 2.3 mIU/mL, Ref: 1.8–11.8 mIU/mL) levels. SHBG (sex hormone-binding globulin), DHEA-S (dehydroepiandrosterone sulfate), androstenedione, 17-hydroxyprogesterone, prolactin, and estradiol levels were all within the normal limits. We have no data regarding pelvic ultrasound findings at that time. Hyperandrogenemia and irregular menstruation were attributed to polycystic ovary syndrome by her doctor, and she was advised to take oral contraceptive pills, which she stopped after a month due to pill dysphagia.

### 2.3. Bilateral Ovarian Sertoli–Leydig Cell Tumors (SLCTs)

In 2019, at the age of 18, she presented to the emergency department with bloating and left-sided abdominal pain. Computed tomography (CT) imaging of the abdomen and pelvis showed a massive mass (20.6 cm in its largest dimension), occupying most of the left side of the abdomen, possibly arising from the left ovary, causing severe obstructive phenomena of the iliac vessels and both the ureters, and resulting in significant ascites. Another smaller mass (5.6 cm in its largest dimension) arising from the right ovary was also depicted. Complementary imaging with abdominal magnetic resonance imaging (MRI) and CT of the thorax revealed no evidence of metastatic disease to the abdomen, pelvis, or chest, but showed small bilateral pleural effusions, suggesting Meigs syndrome (pleural effusion, ascites, and benign ovarian fibroma) [46].

**Figure 2:**
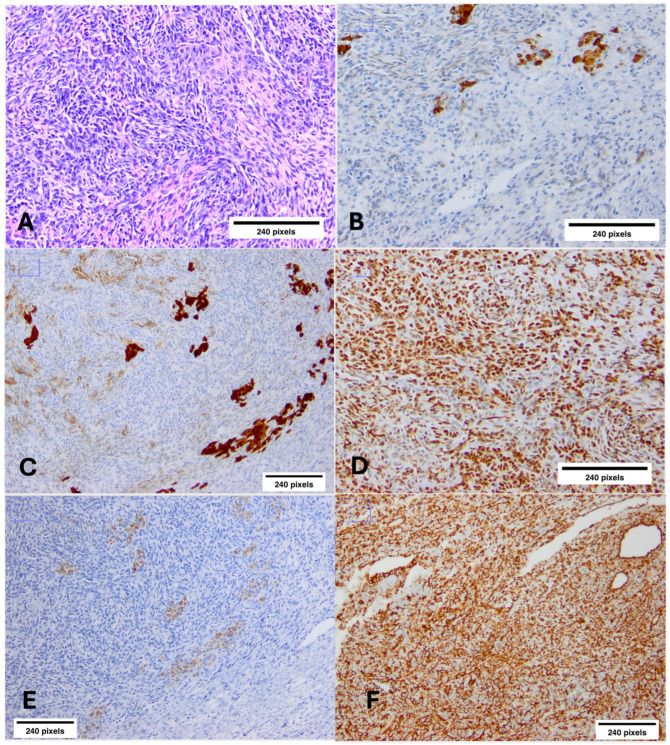
Immunostaining results of moderately differentiated bilateral ovarian Sertoli–Leydig cell tumor (SLCT). (**A**) Diffuse sheet-like pattern (H/E ×10). (**B**) Inhibin-a (×10) highlights Sertoli cells. (**C**) Calretinin (×10) immunostaining reveals positive staining in Sertoli cells. (**D**) WT-1 (×10) immunostaining uniformly highlights the Sertoli cells. (**E**) Melan-A (×10) immunostaining shows weak positivity in Leydig cells. (**F**) Diffuse positivity in Vimentin (×10) staining in SLCT cells (a scale of 240 pixels has been used).

The patient was referred to the gynecology department of our hospital, and the findings were further confirmed by transvaginal and pelvic ultrasonography. She immediately underwent unilateral left oophorectomy and tumor excision of the right ovary. Histology showed both lesions to be moderately differentiated SLCTs. Tumor cells were immunostained for CKAE1/AE3, inhibin, calretinin, WT-1, and focally for melan-A. Any immunohistochemistry (IHC) was indicative of the presence or absence of these proteins, as a quantification method [e.g., immunoblots (Western) for protein quantification] was not performed (Figure 2). No capsular tearing was documented; 15 left parametrial lymph nodes were tumor-free. Since cytologic examination of ascitic fluid was negative for malignant cells, tumor stage was IB according to The International Federation of Gynecology and Obstetrics (FIGO) staging system.

Laboratory investigation of tumor markers showed that cancer antigen 125 (Ca 125) and inhibin were markedly elevated (359.7, Ref: <35 U/mL and 443, Ref: 2–80 pg/mL, respectively), whereas alpha-fetoprotein (AFP; 13.7, Ref: <40 ng/mL), carcinoembryonic antigen (CEA; 0.3, Ref: <2.5 ng/mL), and cancer antigen 15–3 (Ca 15–3; 15.4, Ref: ≤30 U/mL) were normal. Notably, Ca 125 and inhibin decreased to normal approximately 20 days after surgery, as did the hormone profile, with testosterone levels returning to normal (0.4 ng/mL, Ref: 0.05–0.52 ng/mL) (Table 3). Following surgery, the patient was referred to the medical oncology department, where she was advised to receive adjuvant chemotherapy after completing fertility preservation therapy. The latter failed due to unsuccessful ovarian stimulation. FIGO stage, intraperitoneal tumor rupture, and possibly tumor size (>5 cm) are significant prognostic factors [47,48]. Due to the FIGO stage (>IA) and the tumor size (20.6 cm), and according to European Society for Medical Oncology clinical practice guidelines, our patient received three cycles of chemotherapy in total (BEP regimen; bleomycin–etoposide–cisplatin) [41]. Her menstruation cycle returned to normal after the completion of chemotherapy sessions.

### 2.4. Jouvenile Granulosa Cell Tumor (JGCT)

**Figure 3:**
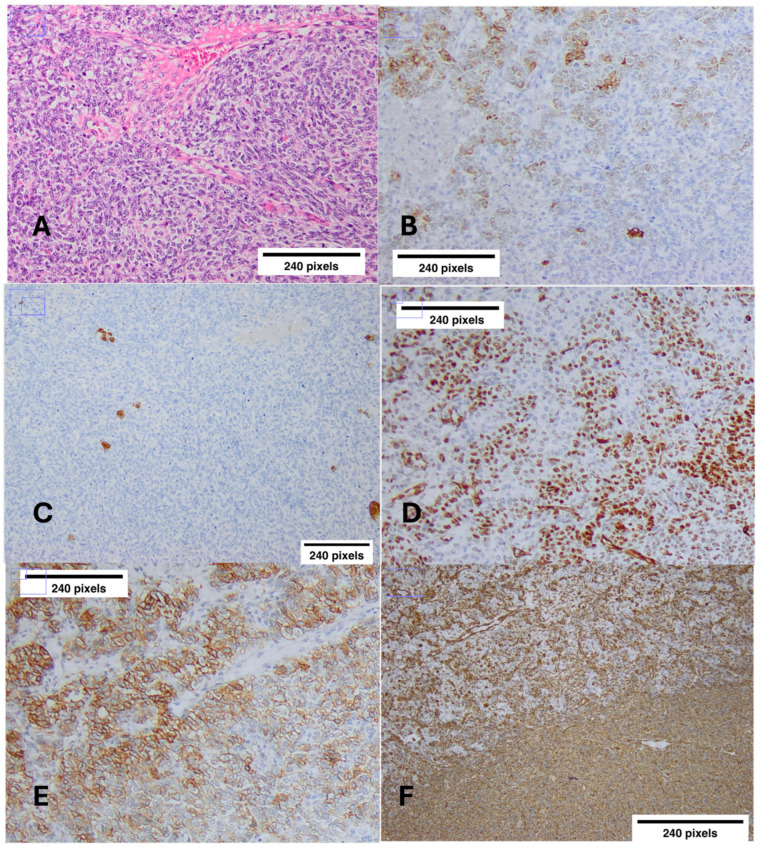
Immunostaining results of juvenile granulosa cell tumor (JGCT) of the right ovary. (**A**) JGCT with diffuse growth pattern, round hyperchromatic nuclei, with small nucleoli and irregular nuclear contours and rare grooves (H/E ×10). (**B**) Inhibin-a (×10) highlights juvenile granulosa cells. (**C**) Calretinin (×10) immunostaining reveals rare immunoreactivity in cells of JGCT. (**D**) WT-1 (×10) immunostaining scattered in JGCT cells. (**E**) CD-99 (×10) reveals positive stain in JGCT cells. (**F**) Diffuse positivity in Vimentin (×10) staining in JGCT cells (a scale of 240 pixels has been used).

Three years postoperatively (2022, 21 years old), during follow-up imaging with abdominal MRI, a large multilocular cystic lesion 13 × 9 × 7.5 cm at the right parametrial area was depicted. Histology showed the excised lesion to be a JGCT; tumor cells were immunostained for CKAE1/AE3 (focally in a “dot-like” juxtanuclear pattern), vimentin, inhibin, CD56, CD99, WT-1, PR, and focally for calretinin (again, any IHC was indicative of the presence or absence of these proteins) (Figure 3). No capsular tearing was documented; seven right pelvic lymph nodes were tumor free. Tumor stage was IA according to the FIGO staging system. Stage IA granulosa cell tumors have an excellent prognosis after surgery alone and do not require adjuvant therapy according to European Society for Medical Oncology clinical practice guidelines [41]. Therefore, the patient was referred to the medical oncology department for follow-up.

### 2.5. Genetic Testing

**Figure 4:**
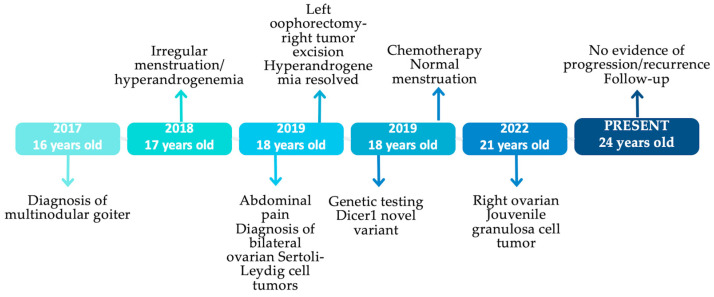
The timeline of our patient’s history.

After her SLCT diagnosis (2019), the patient was referred to a genetic counsellor and genetic testing. Routine and molecular karyotype performed were normal (46, XX). Whole exome sequencing (WES) revealed that she is heterozygous for a new frameshift variant of the *DICER1* gene, in exon 16 (c.2685dupA), consisting of a nucleotide duplication (NM_001195573: c.2685dupA) that is responsible for preterm ending of DICER1 protein synthesis (p. Phe.896IIefs*5) [49]. This variant is characterized as pathogenic according to the criteria of the American College of Medical Genetics and Genomics and the Association for Molecular Pathology [50]. The method of sequencing was next-generation sequencing (NGS), and the variant was confirmed by multiple sequencing and “deep” reads (>100×). The timeline of our patient’s history is depicted in Figure 4.

### 2.6. Family History

Notably, the patient had a positive family history; namely, her maternal aunt had a history of rhabdomyosarcoma of the uterus, diagnosed at the age of 11 years and treated with chemotherapy, and subsequently she was in remission. She also had a history of MNG since the age of 14 years and had undergone total thyroidectomy at the age of 21 years, with no evidence of thyroid carcinoma. Her mother was found to carry the same pathogenic variant, but she was otherwise asymptomatic. To date, her mother refuses to complete the suggested diagnostic workup, and her aunt refuses to be tested for the variant.

## 3. Discussion

Herein, we present the case of a young Greek female patient with DICER1s, consisting of MNG and two sex-cord ovarian tumors, due to a novel pathogenic variant in the *DICER1* gene.

DICER1s is characterized by a high predisposition for the development of a broad spectrum of benign and malignant neoplasms, expressed with a variety of signs and symptoms, depending on the physiopathological mechanisms involved [51]. MNG is a well-known component of the syndrome. Our patient has MNG with benign nodules, based on the ultrasonography and the FNA features, but she has been advised to regular follow-up, as thyroid carcinoma appears in 5–15% of cases [24]. While MNG itself does not necessarily indicate the presence of a *DICER1* pathogenic variant, when combined with SLCT, it is highly suggestive of the syndrome [10]. SLCT is a very rare type of ovarian sex-cord stromal tumor; it nevertheless appears in great frequency among patients with DICER1s. Our patient experienced menstrual irregularities 2 years before the diagnosis and had hyperandrogenemia and suppressed serum FSH and LH levels, abnormalities that were reversed after treatment. The abdominal pain was attributed to mass effect and subsequent obstructive phenomena in the pelvis. Until now, 6 years later, our patient has no evidence of recurrence. JGCT is another type of ovarian sex-cord stromal tumor that has been rarely associated with germline *DICER1* pathogenic variants. DICER1s is most associated with SLCTs, while JGCT has been associated mainly with somatic *DICER1* mutations [52]. There have been two reports of probable germline-associated DICER1s JCGTs: a patient with DICER1-related disorders presented with JGCT at the age of 16 and another patient whose second-degree cousin had pleuropulmonary blastoma developed JGCT at the age of 2 [16]. Our patient presented with an asymptomatic JGCT as a second sex-cord stromal tumor of the ovary that turned out to be stage 1, and until now, she is free of recurrence.

Although most conditions related to DICER1s occur in infancy and childhood, age distribution seems to vary widely. Our patient was diagnosed with a *DICER1* pathogenic variant at the age of 19 years, and her mother is still asymptomatic at the age of 50 years, despite the positive genetic testing. Haley et al. have described a case of a 7-year-old female with SLCT, while the mother has been asymptomatic and not tested for the mutation until she was diagnosed with SLCT at the age of 38 years [40]. Despite the autosomal dominant manner of inheritance, carrying a pathogenic variant of the *DICER1* gene does not always result in the development of the syndrome, indicating its unknown penetrance. It is thought that by the ages of 10 and 50 years, approximately 5% and 19% of patients with a germline *DICER1* pathogenic variant will develop a neoplasm, respectively, with females being at a significantly higher risk than men [53]. Many heterozygous individuals may remain asymptomatic until a second somatic mutation is acquired, involving a crucial codon that affects *DICER1* activity [9,14]. In our case, the patient’s mother, even if she has positive genetic testing, is still asymptomatic, whereas the maternal aunt had rhabdomyosarcoma of the uterus and MNG at the ages of 11 and 14 years, respectively.

Genetic testing for *DICER1* germline pathogenic variants must be offered in all patients with DICER1-related conditions, such as PPB, cystic nephroma, SLCT, cervical embryonal rhabdomyosarcoma, and pituitary and pineal blastoma, with or without positive family history [10,13]. Of course, all first-degree relatives should be screened for the patient’s specific *DICER1* variant. Imaging surveillance recommendations by system have also been suggested, depending on the age and time of diagnosis [13]. When establishing the diagnosis, the significance of genetic counseling and clinical surveillance must be highlighted in order to early recognize and treat complications of the syndrome, not only in patients but also in family members.

To our knowledge, this is the first case associated with DICER1s, consisting of MGN and both types of sex-cord stromal tumors, due to the pathogenic variant in exon 16 c.2685dupA (Phe.896IIefs*5), which, up to date, is a novel pathogenic *DICER1* variant expressed with SLCT, JGCT, and MNG in our patient. Interestingly, the mother is asymptomatic, suggesting the need for further research to elucidate the underlying mechanisms.

## 4. Conclusions

DICER1s is a rare clinical entity predisposing to the development of benign and malignant tumor and nontumorous conditions. Although it is inherited in an autosomal dominant manner, more data is needed to clarify the clinical fingerprint of *DICER1* pathogenic variants, in order to explain symptomatic cases with common or uncommon components of the syndrome with asymptomatic, rather old mothers. Co-occurrence of MNG in childhood with other rare neoplasms, such as SLCT and cystic nephroma, strongly suggests the disorder. Prompt diagnosis is of clinical significance, regarding appropriate monitoring, early recognition and targeted treatment of tumors, identification of other family members and providing proper genetic counselling and testing. There are still unanswered questions regarding the parameters that determine whether particular DICER1s entities are malignant or undergo transformation. In the future, we should focus on developing targeted therapeutic approaches, refining screening protocols to accurately diagnose conditions associated with the syndrome, and improving the identification of at-risk individuals.

## Figures and Tables

**Table 1:** DICER1 syndrome associated phenotypes and frequency.

Most Frequent	Moderate Frequent—Rare	Very Rare
Pleuropulmonary blastoma	Differentiated thyroid cancer	Anaplastic sarcoma of kidney
Multinodular goiter	Wilms tumor	Medulloblastoma
Cystic nephroma	Juvenile hamartomatous intestinal polyps	Embryonal rhabdomyosarcoma of the bladder
Ovarian Sertoli–Leydig cell tumor	Ciliary body medulloepithelioma	Embryonal rhabdomyosarcoma of the ovary
	Nasal chondromesenchymal hamartoma	Neuroblastoma
	Pituitary blastoma	Congenital phthisis bulbi
	Pineoblastoma	Juvenile granulosa cell tumor
	Cervical embryonal rhabdomyosarcoma	Gynandroblastoma
		Cervix primitive neuroectodermal tumor

**Table 2:** DICER1 syndrome associated conditions according to malignant potential.

Neoplastic Conditions			Non-Neoplastic Conditions
Malignant Neoplasms		Benign Neoplasms	
Pleuropulmonary blastoma	Juvenile granulosa cell tumor	Pleuropulmonary blastoma	Macrocephaly
Pineal blastoma	Gynandroblastoma	Multinodular goiter	Kidney structural abnormalities
Ovarian Sertoli–Leydig cell tumor	Embryonal rhabdomyosarcoma (bladder, ovary)	Cystic nephroma	Retinal abnormalities
Cervical embryonal rhabdomyosarcoma	Neuroblastoma	Juvenile hamartomatous intestinal polyps	Dental perturbations
Wilms tumor	Medulloblastoma	Nasal chondromesenchymal hamartoma	GLOW syndrome *
Sarcomas (uterine cervix, kidney, brain)	Ciliary body medulloepithelioma	Ciliary body medulloepithelioma	Congenital phthisis bulbi
Thyroid carcinoma	Mesenchymal hamartoma of the liver		

* GLOW syndrome; global developmental delay, lung cysts, overgrowth, and Wilms tumor.

**Table 3:** Patient’s hormone profile before and after surgery for Sertoli–Leydig cell tumors.

Laboratory Test	Preoperatively	Postoperatively	Reference Range
FSH	1.3	7.6	3–8.1 mIU/mL
LH	2.3	5.8	1.8–11.8 mIU/mL
E2	45	24	21–251 pg/mL
Testosterone	3.1	0.4	0.1–0.5 ng/mL

FSH; follicle-stimulating hormone, LH; luteinizing hormone, E2; estradiol.

## Data Availability

Data are contained within the article.

## Abbreviations

The following abbreviations are used in this manuscript:

AFP	Alpha-fetoprotein
AGO	Argonaute protein
Ca 125	Cancer antigen 125
Ca 15-3	Cancer antigen 15-3
CEA	Carcinoembryonic antigen
CT	Computed tomography
DGCR85	DiGeorge critical region 8
DHEA-S	Dehydroepiandrosterone sulfate
DICER1s	DICER1 syndrome
FIGO	The International Federation of Gynecology and Obstetrics
FNA	Fine needle aspiration
FSH	Follicle-stimulating hormone
GLOW	Global developmental delay, Lung cysts, Overgrowth, Wilms tumor
IHC	Immunohistochemistry
JGCT	Juvenile granulosa cell tumor
LH	Luteinizing hormone
miRNA	Micro RNA
MNG	Multinodular goiter
MRI	Magnetic resonance imaging
NGS	Next-generation sequencing
PPB	Pleuropulmonary blastoma
RISC	RNA-induced silencing complex
SHBG	Sex hormone-binding globulin
SLCT	Sertoli–Leydig cell tumor
TBS	The Bethesda system
WES	Whole exome sequencing
WHO	World Health Organization
